# Bis[6-meth­oxy-2-[(4-methyl­phen­yl)iminiometh­yl]phenolate-κ*O* ^1^]tris­(nitrato-κ^2^ *O*,*O*′)ytterbium(III) monohydrate

**DOI:** 10.1107/S1600536809041361

**Published:** 2009-10-17

**Authors:** Jian-Feng Liu, Jia-Lu Liu, Guo-Liang Zhao

**Affiliations:** a Zhejiang Key Laboratory for Reactive Chemistry on Solid Surfaces, Institute of Physical Chemistry, Zhejiang Normal University, Jinhua, Zhejiang 321004, People’s Republic of China, and College of Chemistry and Life Science, Zhejiang Normal University, Jinhua 321004, Zhejiang, People’s Republic of China

## Abstract

The crystal structure of title compound, [Yb(NO_3_)_3_(C_15_H_15_NO_2_)_2_]·H_2_O, contains two Schiff base 2-[(4-methyl­phen­yl)imino­meth­yl]-6-methoxy­phenol (H*L*) ligands, three independent nitrate ions that chelate to the ytterbium(III) ion in an *O*,*O*′-bidentate manner and an uncoordinated water mol­ecule. The coordination number of the Yb^III^ ion is eight. The H*L* ligands chelate with a strong Yb—O(phenolate) bond and a weak Yb—O(meth­oxy) contact. The latter augments the coordination polyhedron to give a YbO_10_ bicapped square antiprism. Classical inter­molecular O—H⋯O and N—H⋯O hydrogen bonds as well as weak C—H⋯O contacts contribute to the stability of the structure.

## Related literature

For the crystal structure of a zinc(II) complex with two chelating H*L* ligands, see: Xian *et al.* (2008 ▶). For a related terbium(III) complex, see: Zhao *et al.* (2007 ▶). For the zigzag chain cadmium(II) complex bridged by chloride, see: Li *et al.* (2008 ▶). For iron(III) and cobalt(III) complexes of some *N*-salicylideneamino acids, see: Burrows & Bailar (1966 ▶). For a heterodimetallic (Yb, La) complex, see: Costes *et al.* (1998 ▶). For the syntheses of rare earth complexes with Schiff bases derived from *o*-vanillin and adamantaneamine, see: Zhao *et al.* (2005 ▶).
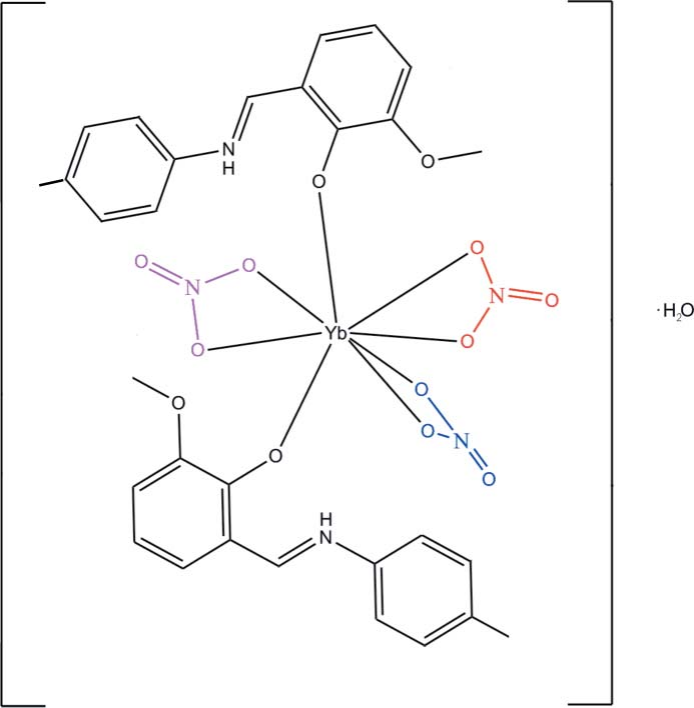


## Experimental

### 

#### Crystal data

[Yb(NO_3_)_3_(C_15_H_15_NO_2_)_2_]·H_2_O
*M* *_r_* = 859.65
Triclinic, 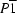

*a* = 9.6878 (1) Å
*b* = 9.9210 (2) Å
*c* = 18.5998 (3) Å
α = 97.341 (1)°
β = 101.929 (1)°
γ = 106.593 (1)°
*V* = 1642.63 (5) Å^3^
*Z* = 2
Mo *K*α radiation
μ = 2.92 mm^−1^
*T* = 296 K
0.27 × 0.16 × 0.10 mm

#### Data collection

Bruker APEXII area-detector diffractometer
Absorption correction: multi-scan (*SADABS*; Sheldrick, 1996 ▶) *T*_min_ = 0.576, *T*_max_ = 0.757
24571 measured reflections
7543 independent reflections
6122 reflections with *I* > 2σ(*I*)
*R*_int_ = 0.029

#### Refinement

*R*[*F* ^2^ > 2σ(*F* ^2^)] = 0.038
*wR*(*F* ^2^) = 0.095
*S* = 0.99
7543 reflections
457 parameters
4 restraints
H atoms treated by a mixture of independent and constrained refinement
Δρ_max_ = 1.88 e Å^−3^
Δρ_min_ = −0.76 e Å^−3^

### 

Data collection: *APEX2* (Bruker, 2006 ▶); cell refinement: *SAINT* (Bruker, 2006 ▶); data reduction: *SAINT*; program(s) used to solve structure: *SHELXS97* (Sheldrick, 2008 ▶); program(s) used to refine structure: *SHELXL97* (Sheldrick, 2008 ▶); molecular graphics: *SHELXTL* (Sheldrick, 2008 ▶); software used to prepare material for publication: *SHELXL97* and *PLATON* (Spek, 2009 ▶).

## Supplementary Material

Crystal structure: contains datablocks I, global. DOI: 10.1107/S1600536809041361/si2199sup1.cif

Structure factors: contains datablocks I. DOI: 10.1107/S1600536809041361/si2199Isup2.hkl

Additional supplementary materials:  crystallographic information; 3D view; checkCIF report

## Figures and Tables

**Table 1:** Selected bond lengths (Å)

Yb—O3	2.225 (3)
Yb—O1	2.228 (3)
Yb—O12	2.342 (3)
Yb—O5	2.373 (3)
Yb—O9	2.379 (4)
Yb—O6	2.404 (3)
Yb—O11	2.444 (4)
Yb—O8	2.451 (4)
Yb—O2	2.833 (4)
Yb—O4	2.927 (3)

**Table 2:** Hydrogen-bond geometry (Å, °)

*D*—H⋯*A*	*D*—H	H⋯*A*	*D*⋯*A*	*D*—H⋯*A*
N1—H1*A*⋯O1	0.86	1.89	2.590 (4)	138
N2—H2*A*⋯O3	0.86	1.99	2.668 (4)	135
O1*W*—H1*WB*⋯O13^i^	0.88	1.89 (13)	2.741	162
O1*W*—H1*WB*⋯N5^i^	0.88	2.55 (11)	3.404	163
O1*W*—H1*WA*⋯O9^ii^	0.88	2.22 (11)	2.97416	144
C22—H22*A*⋯O1*W*	0.93	2.29	3.193	163
C4—H4*A*⋯O7^iii^	0.93	2.45	3.137 (7)	131

Symmetry codes: (i) 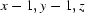; (ii) 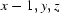; (iii) 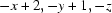.

**Fig. 1.**
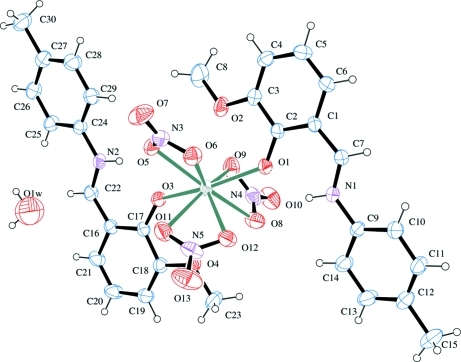
The molecular structure of the title complex, showing the atom-labeling scheme. Displacement ellipsoids are drawn at the 30% probability level.

**Fig. 2.**
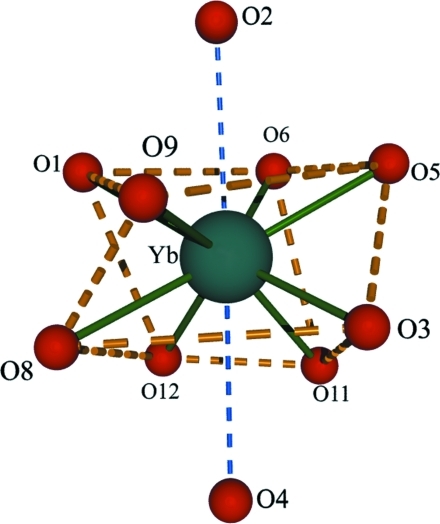
The coordination environment of the Ytterbium(III) atom, showing the bicapped square antiprism.

**Fig. 3.**
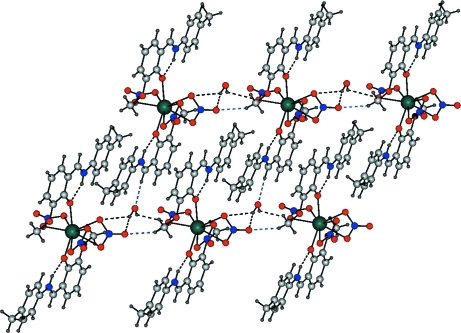
The stacking plot of the title compound, showing H-bond interactions (dashed lines) and π···π stacking interactions.

## supplementary crystallographic information

### Comment

It has well been confirmed that Schiff bases are important in multiple fields such as chemistry and biochemistry owing to their biological activities (Zhao *et al.*, 2005). Schiff base complexes prepared by ligands from substituted *o*-vanillin have been absorbed considerable attention in the past decades due to the intriguing biological activities of *o*-vanillin and the convenience in Schiff bases synthesis (Burrows & Bailar, 1966). Interested in this field, we have been engaged in a major effort directed toward the development of syntheses of new analogous Schiff bases derived from *o*-vanillin and their rare metal complexes. In a few of articles we have reported our partial research results (Zhao *et al.*, 2007; Xian *et al.* 2008; Li *et al.* 2008). Herein, we describe a new ytterbium(III) complex.

The structure of the title complex is shown in Fig.1, and the coordination environment of Yb^III^ is shown in Fig. 2. In this complex the Yb^III^ is eight-coordinated by O atoms, six of which come from three nitrate ions and two come from the Schiff base ligands (H*L*). The H*L* ligands coordinate to the Yb^III^ ion using oxygen atoms from deprotonated phenolic hydroxyl groups. The ten Yb—O bond distances are listed in Table 1 (including weak Yb—O interactions). The distances between Yb^III^ and methoxyl O atoms (2.833 (4) Å and 2.927 (3) Å for Yb—O2 and Yb—O4) are longer than in similar reported complexes (Costes *et al.*, 1998; Zhao *et al.*, 2007), and even longer than the distances between Yb and Nitrate N, indicating the interactions are weak. In contrast, in the Tb^III^ complex Zhao (2007), the Tb—O (methoxyl) bonds are shorter and stronger (2.731 (2) Å and 2.744 (2) Å), which can be attribute to the ionic radii decrease from Tb^III^ to Yb^III^ due to the lanthanide contraction.

The hydrogen bonds and weak π···π non-covalent interactions lend stability to the structure. The hydrogen bonds are listed in Table 2 and the stacking plot of this compound is shown in Fig. 3. Complex molecules are linked in a chain through water molecules by hydrogen bonds, and different chains are interlocked with benzene rings of Schiff base using π···π stacking. In the H*L* ligands, the proton of the phenolic hydroxyl group is considered to have transferred to the *N*-imine atom, which involving in an intramolecular hydrogen bond (Table 2).

### Experimental

Reagents and solvents used were of commercially available quality and without purified before using. The Schiff base ligand 2-[(4- methylphenyl)iminomethyl]-6-methoxy-phenol was prepared by condensation of *o*-vanillin and *p*-methylaniline with a high yield and which was purified by recrystallization in ethanol. The compound (1) was obtained by adding Yb(NO_3_)_3_ (1 mmol, dissolved in methanol) to *N*-salicylidene-*p*-toluidine (3 mmol) in methanol solution. The mixture solution was stirred at room temperature for 8 h to obtain a purplish red solution. At last, the deposit was filtered out and the solution was kept for evaporating. The red crystal was formed after several days.

### Refinement

The structure was solved by direct methods and successive Fourier difference synthesis. The H atoms bonded to C and N atoms were positioned geometrically and refined using a riding model [aliphatic C—H =0.96 Å, *U*_iso_(H) = 1.5*U*_eq_(C), aromatic C—H = 0.93 Å, *U*_iso_(H) = 1.2*U*_eq_(C), and N—H = 0.86 Å with *U*_iso_(H) = 1.2*U*_eq_(N)]. The H atoms bonded to water O atoms were located in difference Fourier maps and refined with O—H distance restraints of 0.88 (2) and *U*_iso_(H) = 1.5*U*_eq_(O).

### Crystal data

**Table:**

[Yb(NO_3_)_3_(C_15_H_15_NO_2_)_2_]·H_2_O	*Z* = 2
*M**_r_* = 859.65	*F*(000) = 858
Triclinic, *P*1	*D*_x_ = 1.738 Mg m^−^^3^
Hall symbol: -P 1	Mo *K*α radiation, λ = 0.71073 Å
*a* = 9.6878 (1) Å	Cell parameters from 9270 reflections
*b* = 9.9210 (2) Å	θ = 2.2–27.6°
*c* = 18.5998 (3) Å	µ = 2.92 mm^−^^1^
α = 97.341 (1)°	*T* = 296 K
β = 101.929 (1)°	Block, red
γ = 106.593 (1)°	0.27 × 0.16 × 0.10 mm
*V* = 1642.63 (5) Å^3^	

### Data collection

**Table:**

Bruker APEXII area-detector diffractometer	7543 independent reflections
Radiation source: fine-focus sealed tube	6122 reflections with *I* > 2σ(*I*)
graphite	*R*_int_ = 0.029
φ and ω scans	θ_max_ = 27.6°, θ_min_ = 2.2°
Absorption correction: multi-scan (*SADABS*; Sheldrick, 1996)	*h* = −12→12
*T*_min_ = 0.576, *T*_max_ = 0.757	*k* = −11→12
24571 measured reflections	*l* = −24→24

### Refinement

**Table:**

Refinement on *F*^2^	Primary atom site location: structure-invariant direct methods
Least-squares matrix: full	Secondary atom site location: difference Fourier map
*R*[*F*^2^ > 2σ(*F*^2^)] = 0.038	Hydrogen site location: inferred from neighbouring sites
*wR*(*F*^2^) = 0.095	H atoms treated by a mixture of independent and constrained refinement
*S* = 0.99	*w* = 1/[σ^2^(*F*_o_^2^) + (0.0562*P*)^2^ + 0.3718*P*] where *P* = (*F*_o_^2^ + 2*F*_c_^2^)/3
7543 reflections	(Δ/σ)_max_ = 0.001
457 parameters	Δρ_max_ = 1.88 e Å^−^^3^
4 restraints	Δρ_min_ = −0.76 e Å^−^^3^

### Special details

**Table:**

Geometry. All esds (except the esd in the dihedral angle between two l.s. planes) are estimated using the full covariance matrix. The cell esds are taken into account individually in the estimation of esds in distances, angles and torsion angles; correlations between esds in cell parameters are only used when they are defined by crystal symmetry. An approximate (isotropic) treatment of cell esds is used for estimating esds involving l.s. planes.
Refinement. Refinement of *F*^2^ against ALL reflections. The weighted *R*-factor *wR* and goodness of fit *S* are based on *F*^2^, conventional *R*-factors *R* are based on *F*, with *F* set to zero for negative *F*^2^. The threshold expression of *F*^2^ > σ(*F*^2^) is used only for calculating *R*-factors(gt) *etc*. and is not relevant to the choice of reflections for refinement. *R*-factors based on *F*^2^ are statistically about twice as large as those based on *F*, and *R*-factors based on ALL data will be even larger.

### Fractional atomic coordinates and isotropic or equivalent isotropic displacement parameters (Å^2^)

**Table:**

	*x*	*y*	*z*	*U*_iso_*/*U*_eq_	
Yb	0.968710 (17)	0.805760 (19)	0.249140 (10)	0.04617 (8)	
N1	1.4545 (4)	1.0479 (4)	0.27759 (19)	0.0459 (8)	
H1A	1.3655	1.0118	0.2820	0.055*	
N2	0.5165 (4)	0.4795 (4)	0.22488 (19)	0.0456 (8)	
H2A	0.6045	0.5268	0.2226	0.055*	
N3	0.8386 (5)	0.7186 (5)	0.0937 (2)	0.0606 (10)	
N4	1.1154 (5)	0.7510 (6)	0.3852 (3)	0.0664 (12)	
N5	0.9364 (6)	1.0733 (5)	0.2322 (3)	0.0681 (12)	
O1	1.2019 (3)	0.8427 (3)	0.24032 (17)	0.0530 (8)	
O2	1.0387 (3)	0.5841 (4)	0.1676 (2)	0.0643 (9)	
O3	0.7601 (3)	0.7075 (3)	0.28207 (17)	0.0517 (7)	
O4	0.8875 (3)	0.9622 (4)	0.36941 (19)	0.0654 (9)	
O5	0.7806 (3)	0.6609 (4)	0.14198 (19)	0.0614 (9)	
O6	0.9582 (4)	0.8237 (4)	0.12055 (19)	0.0621 (8)	
O7	0.7843 (6)	0.6766 (5)	0.0273 (2)	0.0980 (14)	
O8	1.1297 (5)	0.8765 (5)	0.3772 (2)	0.0729 (10)	
O9	1.0374 (4)	0.6518 (4)	0.3277 (2)	0.0716 (10)	
O10	1.1715 (5)	0.7193 (5)	0.4431 (2)	0.0904 (13)	
O11	0.8255 (4)	0.9634 (4)	0.2118 (2)	0.0700 (9)	
O12	1.0607 (4)	1.0550 (3)	0.2605 (2)	0.0611 (9)	
O13	0.9282 (6)	1.1912 (5)	0.2263 (3)	0.1144 (18)	
C1	1.3997 (4)	0.8384 (5)	0.1834 (2)	0.0429 (9)	
C2	1.2576 (4)	0.7763 (5)	0.1938 (2)	0.0439 (9)	
C3	1.1733 (5)	0.6369 (5)	0.1516 (2)	0.0480 (10)	
C4	1.2283 (5)	0.5683 (5)	0.1010 (3)	0.0554 (11)	
H4A	1.1713	0.4774	0.0732	0.067*	
C5	1.3693 (6)	0.6341 (5)	0.0912 (3)	0.0600 (12)	
H5A	1.4049	0.5870	0.0563	0.072*	
C6	1.4543 (5)	0.7647 (5)	0.1316 (2)	0.0527 (11)	
H6A	1.5490	0.8065	0.1255	0.063*	
C7	1.4944 (5)	0.9742 (5)	0.2276 (2)	0.0467 (10)	
H7A	1.5889	1.0115	0.2203	0.056*	
C8	0.9538 (7)	0.4397 (6)	0.1344 (4)	0.092 (2)	
H8A	1.0138	0.3791	0.1452	0.139*	
H8B	0.9214	0.4305	0.0811	0.139*	
H8C	0.8685	0.4114	0.1544	0.139*	
C9	1.5409 (5)	1.1810 (4)	0.3256 (2)	0.0456 (9)	
C10	1.6779 (5)	1.2602 (5)	0.3190 (3)	0.0576 (11)	
H10A	1.7168	1.2282	0.2810	0.069*	
C11	1.7572 (5)	1.3869 (5)	0.3689 (3)	0.0619 (12)	
H11A	1.8502	1.4395	0.3643	0.074*	
C12	1.7019 (6)	1.4379 (5)	0.4256 (3)	0.0603 (12)	
C13	1.5647 (6)	1.3591 (6)	0.4309 (3)	0.0721 (15)	
H13A	1.5257	1.3921	0.4686	0.087*	
C14	1.4825 (6)	1.2312 (6)	0.3812 (3)	0.0673 (14)	
H14A	1.3888	1.1795	0.3853	0.081*	
C15	1.7903 (7)	1.5764 (6)	0.4809 (3)	0.0851 (18)	
H15A	1.7900	1.5618	0.5309	0.128*	
H15B	1.7462	1.6492	0.4705	0.128*	
H15C	1.8910	1.6061	0.4764	0.128*	
C16	0.5417 (4)	0.6699 (5)	0.3259 (2)	0.0442 (9)	
C17	0.6855 (4)	0.7534 (5)	0.3255 (2)	0.0424 (9)	
C18	0.7486 (5)	0.8889 (5)	0.3747 (2)	0.0481 (10)	
C19	0.6746 (5)	0.9362 (5)	0.4222 (3)	0.0569 (11)	
H19A	0.7185	1.0252	0.4544	0.068*	
C20	0.5325 (6)	0.8503 (6)	0.4224 (3)	0.0696 (15)	
H20A	0.4826	0.8825	0.4550	0.083*	
C21	0.4670 (5)	0.7214 (5)	0.3758 (3)	0.0602 (12)	
H21A	0.3720	0.6658	0.3762	0.072*	
C22	0.4648 (5)	0.5359 (5)	0.2758 (2)	0.0476 (10)	
H22A	0.3708	0.4851	0.2798	0.057*	
C23	0.9660 (6)	1.0991 (5)	0.4183 (3)	0.0681 (14)	
H23A	0.9765	1.0881	0.4695	0.102*	
H23B	0.9111	1.1644	0.4085	0.102*	
H23C	1.0628	1.1363	0.4097	0.102*	
C24	0.4433 (4)	0.3477 (4)	0.1723 (2)	0.0443 (9)	
C25	0.3097 (5)	0.2547 (5)	0.1744 (3)	0.0531 (11)	
H25A	0.2642	0.2759	0.2118	0.064*	
C26	0.2433 (5)	0.1309 (5)	0.1216 (3)	0.0589 (12)	
H26A	0.1524	0.0688	0.1235	0.071*	
C27	0.3079 (6)	0.0956 (5)	0.0654 (3)	0.0590 (12)	
C28	0.4427 (6)	0.1890 (6)	0.0646 (3)	0.0708 (14)	
H28A	0.4887	0.1667	0.0277	0.085*	
C29	0.5122 (5)	0.3158 (6)	0.1174 (3)	0.0628 (13)	
H29A	0.6033	0.3780	0.1158	0.075*	
C30	0.2304 (5)	−0.0412 (5)	0.0060 (3)	0.0803 (16)	
H30A	0.1376	−0.0919	0.0155	0.121*	
H30B	0.2930	−0.1009	0.0077	0.121*	
H30C	0.2117	−0.0171	−0.0427	0.121*	
O1W	0.1609 (5)	0.4096 (5)	0.3221 (3)	0.207 (4)	
H1WB	0.086 (11)	0.330 (10)	0.300 (8)	0.310*	
H1WA	0.117 (14)	0.466 (10)	0.342 (5)	0.310*	

### Atomic displacement parameters (Å^2^)

**Table:**

	*U*^11^	*U*^22^	*U*^33^	*U*^12^	*U*^13^	*U*^23^
Yb	0.03135 (10)	0.04494 (13)	0.05706 (13)	0.00384 (8)	0.01277 (8)	0.00902 (9)
N1	0.0345 (17)	0.047 (2)	0.050 (2)	0.0088 (15)	0.0070 (15)	0.0024 (16)
N2	0.0332 (17)	0.049 (2)	0.050 (2)	0.0065 (15)	0.0106 (15)	0.0092 (16)
N3	0.062 (3)	0.061 (3)	0.058 (3)	0.026 (2)	0.011 (2)	0.002 (2)
N4	0.056 (3)	0.094 (4)	0.062 (3)	0.032 (3)	0.027 (2)	0.021 (3)
N5	0.089 (3)	0.061 (3)	0.082 (3)	0.039 (3)	0.051 (3)	0.029 (2)
O1	0.0361 (15)	0.061 (2)	0.0549 (17)	0.0093 (13)	0.0141 (13)	−0.0023 (15)
O2	0.0433 (17)	0.055 (2)	0.082 (2)	−0.0006 (15)	0.0122 (16)	0.0132 (18)
O3	0.0392 (15)	0.0500 (18)	0.0610 (18)	0.0054 (13)	0.0213 (14)	0.0007 (14)
O4	0.0477 (18)	0.059 (2)	0.072 (2)	−0.0028 (15)	0.0197 (16)	−0.0090 (17)
O5	0.0461 (17)	0.063 (2)	0.064 (2)	0.0046 (15)	0.0174 (15)	−0.0037 (17)
O6	0.062 (2)	0.055 (2)	0.066 (2)	0.0109 (17)	0.0205 (17)	0.0134 (17)
O7	0.130 (4)	0.096 (3)	0.050 (2)	0.036 (3)	−0.003 (2)	−0.004 (2)
O8	0.086 (3)	0.077 (3)	0.064 (2)	0.042 (2)	0.0200 (19)	0.008 (2)
O9	0.056 (2)	0.069 (2)	0.089 (3)	0.0118 (18)	0.0153 (19)	0.034 (2)
O10	0.096 (3)	0.136 (4)	0.064 (2)	0.064 (3)	0.025 (2)	0.042 (3)
O11	0.058 (2)	0.068 (2)	0.091 (3)	0.0236 (19)	0.0271 (19)	0.019 (2)
O12	0.059 (2)	0.0428 (18)	0.087 (2)	0.0130 (15)	0.0361 (18)	0.0134 (17)
O13	0.159 (5)	0.073 (3)	0.159 (5)	0.065 (3)	0.085 (4)	0.056 (3)
C1	0.0340 (19)	0.048 (2)	0.046 (2)	0.0135 (18)	0.0083 (17)	0.0095 (19)
C2	0.037 (2)	0.050 (3)	0.043 (2)	0.0160 (18)	0.0060 (17)	0.0066 (19)
C3	0.039 (2)	0.050 (3)	0.050 (2)	0.0103 (19)	0.0054 (18)	0.011 (2)
C4	0.062 (3)	0.041 (3)	0.055 (3)	0.013 (2)	0.005 (2)	0.002 (2)
C5	0.068 (3)	0.059 (3)	0.057 (3)	0.027 (3)	0.020 (2)	0.002 (2)
C6	0.048 (2)	0.060 (3)	0.052 (3)	0.021 (2)	0.017 (2)	0.005 (2)
C7	0.039 (2)	0.052 (3)	0.047 (2)	0.0164 (19)	0.0076 (18)	0.003 (2)
C8	0.069 (4)	0.052 (3)	0.143 (6)	−0.001 (3)	0.019 (4)	0.031 (4)
C9	0.044 (2)	0.040 (2)	0.047 (2)	0.0105 (18)	0.0046 (18)	0.0055 (19)
C10	0.049 (3)	0.053 (3)	0.068 (3)	0.010 (2)	0.020 (2)	0.007 (2)
C11	0.051 (3)	0.045 (3)	0.077 (3)	0.001 (2)	0.011 (2)	0.007 (2)
C12	0.063 (3)	0.043 (3)	0.060 (3)	0.006 (2)	−0.003 (2)	0.011 (2)
C13	0.082 (4)	0.055 (3)	0.067 (3)	0.009 (3)	0.026 (3)	−0.010 (3)
C14	0.060 (3)	0.055 (3)	0.075 (3)	0.003 (2)	0.025 (3)	−0.004 (3)
C15	0.098 (5)	0.049 (3)	0.076 (4)	0.003 (3)	−0.004 (3)	−0.007 (3)
C16	0.042 (2)	0.046 (2)	0.048 (2)	0.0147 (18)	0.0151 (18)	0.0130 (19)
C17	0.037 (2)	0.047 (2)	0.045 (2)	0.0138 (18)	0.0128 (17)	0.0111 (19)
C18	0.044 (2)	0.050 (3)	0.049 (2)	0.0122 (19)	0.0130 (19)	0.009 (2)
C19	0.059 (3)	0.050 (3)	0.064 (3)	0.020 (2)	0.020 (2)	0.006 (2)
C20	0.073 (3)	0.062 (3)	0.083 (4)	0.022 (3)	0.046 (3)	0.003 (3)
C21	0.052 (3)	0.060 (3)	0.076 (3)	0.017 (2)	0.036 (2)	0.011 (3)
C22	0.041 (2)	0.045 (2)	0.057 (3)	0.0079 (18)	0.0181 (19)	0.017 (2)
C23	0.060 (3)	0.054 (3)	0.070 (3)	0.000 (2)	0.008 (2)	−0.004 (3)
C24	0.043 (2)	0.039 (2)	0.048 (2)	0.0096 (18)	0.0101 (18)	0.0102 (18)
C25	0.049 (2)	0.049 (3)	0.055 (3)	0.004 (2)	0.017 (2)	0.009 (2)
C26	0.051 (3)	0.051 (3)	0.065 (3)	0.001 (2)	0.015 (2)	0.015 (2)
C27	0.061 (3)	0.052 (3)	0.056 (3)	0.014 (2)	0.004 (2)	0.011 (2)
C28	0.064 (3)	0.070 (4)	0.076 (3)	0.014 (3)	0.029 (3)	0.002 (3)
C29	0.045 (3)	0.061 (3)	0.076 (3)	0.007 (2)	0.025 (2)	0.001 (3)
C30	0.083 (4)	0.060 (3)	0.077 (4)	0.012 (3)	0.001 (3)	−0.004 (3)
O1W	0.172 (7)	0.191 (9)	0.248 (10)	0.041 (6)	0.078 (7)	0.018 (7)

### Geometric parameters (Å, °)

**Table:**

Yb—O3	2.225 (3)	C8—H8B	0.9600
Yb—O1	2.228 (3)	C8—H8C	0.9600
Yb—O12	2.342 (3)	C9—C10	1.375 (6)
Yb—O5	2.373 (3)	C9—C14	1.383 (6)
Yb—O9	2.379 (4)	C10—C11	1.373 (6)
Yb—O6	2.404 (3)	C10—H10A	0.9300
Yb—O11	2.444 (4)	C11—C12	1.382 (7)
Yb—O8	2.451 (4)	C11—H11A	0.9300
Yb—N5	2.809 (5)	C12—C13	1.367 (7)
Yb—N3	2.815 (4)	C12—C15	1.510 (7)
Yb—O2	2.833 (4)	C13—C14	1.384 (7)
Yb—O4	2.927 (3)	C13—H13A	0.9300
Yb—N4	2.833 (5)	C14—H14A	0.9300
N1—C7	1.298 (5)	C15—H15A	0.9600
N1—C9	1.414 (5)	C15—H15B	0.9600
N1—H1A	0.8600	C15—H15C	0.9600
N2—C22	1.297 (5)	C16—C17	1.405 (5)
N2—C24	1.423 (5)	C16—C21	1.419 (6)
N2—H2A	0.8600	C16—C22	1.423 (6)
N3—O7	1.205 (5)	C17—C18	1.416 (6)
N3—O5	1.267 (5)	C18—C19	1.365 (6)
N3—O6	1.271 (5)	C19—C20	1.397 (7)
N4—O10	1.223 (6)	C19—H19A	0.9300
N4—O8	1.245 (6)	C20—C21	1.347 (7)
N4—O9	1.287 (6)	C20—H20A	0.9300
N5—O13	1.212 (6)	C21—H21A	0.9300
N5—O11	1.242 (6)	C22—H22A	0.9300
N5—O12	1.284 (6)	C23—H23A	0.9600
O1—C2	1.313 (5)	C23—H23B	0.9600
O2—C3	1.369 (5)	C23—H23C	0.9600
O2—C8	1.415 (6)	C24—C25	1.369 (5)
O3—C17	1.310 (5)	C24—C29	1.381 (6)
O4—C18	1.365 (5)	C25—C26	1.367 (6)
O4—C23	1.435 (5)	C25—H25A	0.9300
C1—C2	1.402 (5)	C26—C27	1.381 (7)
C1—C6	1.412 (6)	C26—H26A	0.9300
C1—C7	1.425 (6)	C27—C28	1.373 (7)
C2—C3	1.417 (6)	C27—C30	1.524 (7)
C3—C4	1.372 (6)	C28—C29	1.388 (7)
C4—C5	1.397 (7)	C28—H28A	0.9300
C4—H4A	0.9300	C29—H29A	0.9300
C5—C6	1.344 (6)	C30—H30A	0.9600
C5—H5A	0.9300	C30—H30B	0.9600
C6—H6A	0.9300	C30—H30C	0.9600
C7—H7A	0.9300	O1W—H1WB	0.88
C8—H8A	0.9600	O1W—H1WA	0.88
			
O3—Yb—O1	157.78 (13)	C2—C1—C6	120.7 (4)
O3—Yb—O12	119.91 (11)	C2—C1—C7	120.5 (4)
O1—Yb—O12	77.05 (11)	C6—C1—C7	118.7 (4)
O3—Yb—O5	70.20 (11)	O1—C2—C1	122.4 (4)
O1—Yb—O5	115.39 (10)	O1—C2—C3	120.0 (4)
O12—Yb—O5	120.65 (13)	C1—C2—C3	117.6 (4)
O3—Yb—O9	77.22 (12)	O2—C3—C4	126.4 (4)
O1—Yb—O9	80.67 (12)	O2—C3—C2	113.0 (4)
O12—Yb—O9	131.26 (14)	C4—C3—C2	120.6 (4)
O5—Yb—O9	108.04 (14)	C3—C4—C5	120.4 (4)
O3—Yb—O6	119.78 (11)	C3—C4—H4A	119.8
O1—Yb—O6	75.37 (11)	C5—C4—H4A	119.8
O12—Yb—O6	79.10 (12)	C6—C5—C4	120.8 (4)
O5—Yb—O6	53.28 (11)	C6—C5—H5A	119.6
O9—Yb—O6	135.12 (13)	C4—C5—H5A	119.6
O3—Yb—O11	78.88 (12)	C5—C6—C1	119.9 (4)
O1—Yb—O11	123.06 (12)	C5—C6—H6A	120.0
O12—Yb—O11	53.16 (12)	C1—C6—H6A	120.0
O5—Yb—O11	76.57 (13)	N1—C7—C1	122.9 (4)
O9—Yb—O11	152.18 (13)	N1—C7—H7A	118.6
O6—Yb—O11	70.21 (12)	C1—C7—H7A	118.6
O3—Yb—O8	95.26 (13)	O2—C8—H8A	109.5
O1—Yb—O8	72.75 (12)	O2—C8—H8B	109.5
O12—Yb—O8	79.24 (14)	H8A—C8—H8B	109.5
O5—Yb—O8	159.19 (15)	O2—C8—H8C	109.5
O9—Yb—O8	52.78 (14)	H8A—C8—H8C	109.5
O6—Yb—O8	144.64 (13)	H8B—C8—H8C	109.5
O11—Yb—O8	116.21 (13)	C10—C9—C14	119.7 (4)
O3—Yb—N5	99.59 (12)	C10—C9—N1	123.0 (4)
O1—Yb—N5	100.65 (13)	C14—C9—N1	117.3 (4)
O12—Yb—N5	26.97 (13)	C11—C10—C9	119.8 (4)
O5—Yb—N5	98.56 (14)	C11—C10—H10A	120.1
O9—Yb—N5	149.93 (15)	C9—C10—H10A	120.1
O6—Yb—N5	72.70 (12)	C10—C11—C12	121.5 (4)
O11—Yb—N5	26.20 (13)	C10—C11—H11A	119.3
O8—Yb—N5	98.52 (15)	C12—C11—H11A	119.3
O3—Yb—N3	95.23 (12)	C13—C12—C11	118.2 (4)
O1—Yb—N3	95.27 (12)	C13—C12—C15	120.6 (5)
O12—Yb—N3	100.83 (13)	C11—C12—C15	121.3 (5)
O5—Yb—N3	26.55 (11)	C12—C13—C14	121.5 (5)
O9—Yb—N3	124.08 (14)	C12—C13—H13A	119.3
O6—Yb—N3	26.74 (11)	C14—C13—H13A	119.3
O11—Yb—N3	72.08 (12)	C9—C14—C13	119.4 (5)
O8—Yb—N3	167.78 (12)	C9—C14—H14A	120.3
N5—Yb—N3	85.88 (13)	C13—C14—H14A	120.3
O3—Yb—O2	108.26 (10)	C12—C15—H15A	109.5
O1—Yb—O2	60.78 (10)	C12—C15—H15B	109.5
O12—Yb—O2	130.25 (10)	H15A—C15—H15B	109.5
O5—Yb—O2	63.94 (11)	C12—C15—H15C	109.5
O9—Yb—O2	68.91 (13)	H15A—C15—H15C	109.5
O6—Yb—O2	66.31 (11)	H15B—C15—H15C	109.5
O11—Yb—O2	133.11 (12)	C17—C16—C21	119.8 (4)
O8—Yb—O2	109.30 (11)	C17—C16—C22	122.1 (4)
N5—Yb—O2	137.96 (11)	C21—C16—C22	118.0 (4)
N3—Yb—O2	61.23 (11)	O3—C17—C16	121.6 (4)
O3—Yb—N4	85.22 (12)	O3—C17—C18	120.8 (3)
O1—Yb—N4	75.93 (11)	C16—C17—C18	117.5 (4)
O12—Yb—N4	105.01 (15)	C19—C18—O4	125.4 (4)
O5—Yb—N4	134.23 (15)	C19—C18—C17	121.6 (4)
O9—Yb—N4	26.82 (13)	O4—C18—C17	113.0 (4)
O6—Yb—N4	149.20 (11)	C18—C19—C20	119.8 (4)
O11—Yb—N4	136.87 (13)	C18—C19—H19A	120.1
O8—Yb—N4	25.98 (13)	C20—C19—H19A	120.1
N5—Yb—N4	123.86 (15)	C21—C20—C19	120.8 (4)
N3—Yb—N4	149.85 (14)	C21—C20—H20A	119.6
O2—Yb—N4	89.92 (12)	C19—C20—H20A	119.6
C7—N1—C9	127.4 (4)	C20—C21—C16	120.4 (4)
C7—N1—H1A	116.3	C20—C21—H21A	119.8
C9—N1—H1A	116.3	C16—C21—H21A	119.8
C22—N2—C24	127.0 (3)	N2—C22—C16	124.6 (4)
C22—N2—H2A	116.5	N2—C22—H22A	117.7
C24—N2—H2A	116.5	C16—C22—H22A	117.7
O7—N3—O5	122.4 (5)	O4—C23—H23A	109.5
O7—N3—O6	122.6 (5)	O4—C23—H23B	109.5
O5—N3—O6	115.1 (4)	H23A—C23—H23B	109.5
O7—N3—Yb	177.5 (4)	O4—C23—H23C	109.5
O5—N3—Yb	56.8 (2)	H23A—C23—H23C	109.5
O6—N3—Yb	58.3 (2)	H23B—C23—H23C	109.5
O10—N4—O8	123.9 (5)	C25—C24—C29	120.2 (4)
O10—N4—O9	120.0 (5)	C25—C24—N2	122.6 (4)
O8—N4—O9	116.0 (4)	C29—C24—N2	117.2 (4)
O10—N4—Yb	175.9 (4)	C26—C25—C24	120.0 (4)
O8—N4—Yb	59.6 (3)	C26—C25—H25A	120.0
O9—N4—Yb	56.5 (2)	C24—C25—H25A	120.0
O13—N5—O11	122.2 (5)	C25—C26—C27	121.6 (4)
O13—N5—O12	121.7 (5)	C25—C26—H26A	119.2
O11—N5—O12	116.1 (4)	C27—C26—H26A	119.2
O13—N5—Yb	177.5 (5)	C28—C27—C26	117.7 (5)
O11—N5—Yb	60.3 (2)	C28—C27—C30	121.5 (5)
O12—N5—Yb	55.8 (2)	C26—C27—C30	120.8 (4)
C2—O1—Yb	131.5 (3)	C27—C28—C29	121.9 (5)
C3—O2—C8	117.1 (4)	C27—C28—H28A	119.1
C3—O2—Yb	110.7 (3)	C29—C28—H28A	119.1
C8—O2—Yb	131.7 (3)	C24—C29—C28	118.6 (4)
C17—O3—Yb	135.2 (3)	C24—C29—H29A	120.7
C18—O4—C23	118.6 (4)	C28—C29—H29A	120.7
C18—O4—Yb	110.7 (2)	C27—C30—H30A	109.5
C23—O4—Yb	130.7 (3)	C27—C30—H30B	109.5
N3—O5—Yb	96.6 (3)	H30A—C30—H30B	109.5
N3—O6—Yb	95.0 (3)	C27—C30—H30C	109.5
N4—O8—Yb	94.4 (3)	H30A—C30—H30C	109.5
N4—O9—Yb	96.7 (3)	H30B—C30—H30C	109.5
N5—O11—Yb	93.5 (3)	H1WB—O1W—H1WA	103
N5—O12—Yb	97.2 (3)		

### Hydrogen-bond geometry (Å, °)

**Table:**

*D*—H···*A*	*D*—H	H···*A*	*D*···*A*	*D*—H···*A*
N1—H1A···O1	0.86	1.89	2.590 (4)	138
N2—H2A···O3	0.86	1.99	2.668 (4)	135
O1W—H1WB···O13^i^	0.88	1.89 (13)	2.74133	162
O1W—H1WB···N5^i^	0.88	2.55 (11)	3.40359	163
O1W—H1WA···O9^ii^	0.88	2.22 (11)	2.97416	144
C22—H22A···O1W	0.93	2.29	3.1932	163
C4—H4A···O7^iii^	0.93	2.45	3.137 (7)	131

Symmetry codes: (i) *x*−1, *y*−1, *z*; (ii) *x*−1, *y*, *z*; (iii) −*x*+2, −*y*+1, −*z*.
